# A Delphi study to guide the development of a clinical indicator tool for palliative care in South Africa

**DOI:** 10.4102/phcfm.v14i1.3351

**Published:** 2022-05-19

**Authors:** Rene Krause, Alan Barnard, Henriette Burger, Andre de Vos, Katya Evans, Lindsay Farrant, Nicki Fouche, Sebastiana Kalula, Jennie Morgan, Zainab Mohamed, Eugenio Panieri, Tasleem Ras, Peter Raubenheimer, Estelle Verburg, Kirsty Boyd, Liz Gwyther

**Affiliations:** 1 Department of Family Medicine and Public Health, Faculty of Health Sciences, University of Cape Town, Cape Town, South Africa; 2 Department of Oncology, Faculty of Health Sciences, Stellenbosch University, Cape Town, South Africa; 3 Department of Social Work, Groote Schuur Hospital, Cape Town, South Africa; 4 Department of Emergency Medicine, Faculty of Health Sciences, University of Cape Town, South Africa; 5 Department of Nursing, Faculty of Health Sciences, University of Cape Town, Cape Town, South Africa; 6 Department of Medicine, Faculty of Health Sciences, University of Cape Town, Cape Town, South Africa; 7 Department of Oncology, Faculty of Health Sciences, University of Cape Town, Cape Town, South Africa; 8 Department of General Surgery, Faculty of Health Sciences, University of Cape Town, Cape Town, South Africa; 9 Usher Institute, Primary Palliative Care, University of Edinburgh, Edinburgh, United Kingdom; 10 Department of Family Medicine, School of Public Health and Family Medicine, University of Cape Town, Cape Town, South Africa

**Keywords:** palliative care, indicator tool, Delphi study, trauma, infectious diseases, haematology

## Abstract

**Background:**

The South African National Policy Framework and Strategy on Palliative Care (NPFSPC) recommends that when integrating palliative care (PC) into the health system, a PC indicators tool should be used to guide clinicians to recognise a patient who should receive PC. The policy document recommends ‘a simple screening tool developed for use in South Africa that would assist healthcare professionals (HCPs) to recognise patients who may have unmet palliative care needs’.

**Aim:**

This research study sought to develop South African consensus on indicators for PC to assist clinicians to recognise a patient in need of PC.

**Setting:**

The South African healthcare setting.

**Methods:**

A Delphi study was considered suitable as a methodology to develop consensus. The methodology was based on the Conducting and REporting of DElphi studies (CREDES) guidance on Delphi studies to ensure rigour and transparency in conducting and reporting. Six different Delphi rounds were used to develop consensus. Each round allowed participants to anonymously rate statements with predefined rating scales.

**Results:**

Cognisant of the disparities in healthcare provision and access to equitable healthcare in South Africa, the expert advisory group recommended, especially for South Africa, that ‘this tool is for deteriorating patients with an advanced life-limiting illness where all available and appropriate management for underlying illnesses and reversible complications has been offered’. The expert advisory group felt that disease-specific indicators should be described before the general indicators in the South African indicators tool, so all users of the tool orientate themselves to the disease categories first. This study included three new domains to address the South African context: trauma, infectious diseases and haematological diseases. General indicators for PC aligned with the original Supportive and Palliative Care Indicators Tool (SPICT) tool.

**Conclusion:**

The Supportive and Palliative Care Indicators Tool for South Africa (SPICT^TM^-SA) is a simple screening tool for South Africa that may assist HCPs to recognise patients who may have unmet PC needs.

## Background

The World Health Assembly 67.19 resolution on palliative care (PC) states that the provision of PC is ‘an ethical responsibility of health systems’, and it is the ‘duty of healthcare professionals (HCPs) to alleviate pain and suffering’.^1^ In the Global Atlas of PC, the World Hospice Palliative Care Alliance (WHPCA) estimates that globally, 56.8 million people require PC every year, but PC is not accessible in many low- or middle-income countries (LMICs).^2^ One of the barriers to PC access is the failure to recognise that a patient may benefit from PC.^3^ The European Association of Palliative Care (EAPC) Task Force on Primary PC identified that 1% of a country’s population in Europe is living with advanced chronic disease and would benefit from having a PC approach integrated with their current care. The EAPC estimated that this group accounted for 75% of deaths and one-third of health system expenditure.^4^ The South African National Policy Framework and Strategy on Palliative Care (NPFSPC) recommends that when integrating PC into the health system, a PC indicators tool should be used. This tool aims to guide doctors and other clinicians to recognise that a patient who should receive PC.^5^

The NPFSPC policy document recommends ‘a simple screening tool developed for use in South Africa that would assist HCP to recognise patients who may have unmet PC needs’ and identified the Supportive and Palliative Care Indicators Tool (SPICT^TM^) and the Gold Standards Framework Prognostic Indicator Guidance (GSF-PIG) as two such validated tools, which comprise disease-specific indicators and general indicators of a progressive illness and help to identify a need for PC.^6,7^

The World Health Organization definition of PC clearly states that PC ‘is applicable early in the course of illness, in conjunction with other therapies that are intended to prolong life’.^8^ A PC approach should therefore be considered whenever patients are diagnosed with life-threatening illnesses. The health system, including the HCP involved, often thinks of PC as applicable only at the very end of life, that is, the *dying phase* of an illness. This late recognition of the need for PC denies patients and families the physical, emotional and spiritual care needed as the end-of-life approaches and may hinder discussion about goals of care and care planning.^3^ This results in confusion in all role players as to when to initiate PC. The SPICT^TM^ developed at the University of Edinburgh was designed to assist clinicians to identify people needing PC. The SPICT^TM^ uses easily identifiable clinical indicators of increasing burden of illness and dependence to prompt clinicians to integrate a PC approach into usual care for the patients and to consider referral for specialist PC consultation if the patient’s needs warrant this.

The utility and validity of PC indicator tools developed and used in other parts of the world may be limited for the South African context because of differences in the socio-economic environments and the availability of resources for healthcare. South Africa has extreme socio-economic inequality, evidenced by the high Gini coefficient.^9^ Scarce resources limit access to medical care with 84% of the population relying on an overburdened and underfunded state health system, which cannot provide care in line with the private healthcare system, which runs in parallel.^10^ South Africa also has high mortality because of infectious diseases and trauma.^11^ Considering the economic inequality, the distinctive disease profile and the need for equitable care, there is a need to develop consensus on a simple screening tool to identify unmet PC needs in South Africa.

This research study aimed to develop the South African consensus on indicators for PC to assist clinicians to recognise a patient in need of PC. The objectives of the study were to identify panellists who were representative of the South African healthcare context, to ask them to respond to a survey of possible indicators for PC, to come to a consensus through a Delphi process and to have an external group review the consensus document

## Methods

**FIGURE 1:**
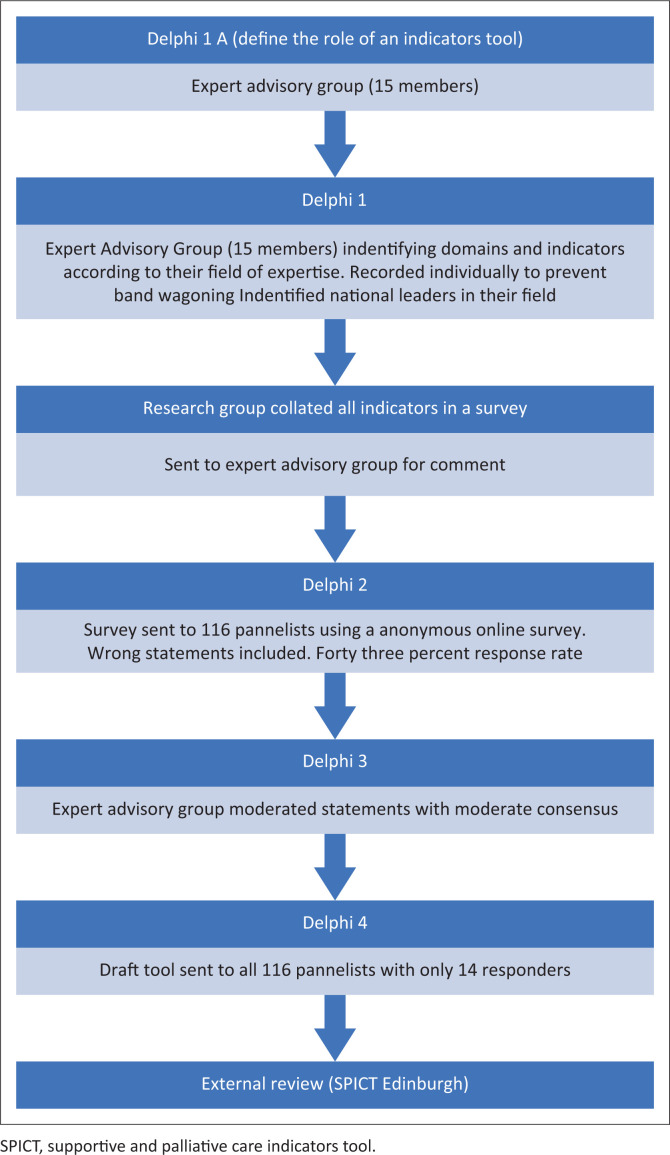
Flow diagram of the study methodology using the Delphi approach.

A Delphi study (Figure 1) was considered suitable as a methodology to develop consensus around indicators for PC in South Africa. To ensure rigour and transparency in the conduct and reporting of this study, its methodology was based on the Conducting and REporting of DElphi studies (CREDE) guidance on Delphi studies.^12^

By applying the CREDE tool, bandwagoning was prevented, and anonymity was ensured by allowing the expert group to provide individual input and by using an anonymous online survey tool (Research Electronic Data Capture [REDCAP]) for the panellist. Rating scales were predefined and the definition of consensus was predefined for all the Delphi rounds. Consensus was defined when ≥ 80% of the participants rated a statement as ‘agreed’ or ‘strongly agreed’. Statements with ‘moderate’ to ‘low agreement’ were fed to the next Delphi round. If no consensus was reached and arbitrator from outside would be appointed.

A research team consisting of three PC practitioners with more than 15 years of experience in the field convened an expert group. The expert advisory group was formed consisting of South African healthcare workers who were deemed experts because they had more than five years of experience in their field, they were trained in PC and regularly referred to PC. This group included three PC clinicians, one general surgeon, two specialist physicians, two specialist haematologists, one social worker, two oncologists, two family physicians, one emergency medicine specialist, one registered nurse and one specialist geriatrician.

### Ethical considerations

Ethics approval was received from University of Cape Town Human Research Ethics Committee (HREC 750/2018 and R012/2016).

## Results

The expert advisory group met face-to-face to reach a consensus on the role of a PC indicators tool for South Africa. This consensus was intended to meet the requirements of the first step in the Delphi process (Delphi 1A).

The initial consensus in January 2019 on the purpose of the tool was that:

- The indicators tool aims to identify adult patients who have serious health-related suffering due to life-limiting or life-threatening illness and whose health is deteriorating. Patients who suffer from such conditions are likely to die as a consequence. This is a generic tool for the South African setting to identify patients who will benefit from a palliative care approach in conjunction with usual care by the treating clinician. This patient may need to be referred to a palliative care team to optimise care.

The expert advisory group members were provided with reference materials describing international indicator tools. They developed a draft survey tool for the first Delphi round by specifying domains of illness and indicators for PC based on their field of expertise. All members also submitted general indicators of deteriorating health, which would indicate a need for PC. In order to preserve independence of contributions, members recorded their thoughts individually and were encouraged not to share these during the initial process. The research team analysed all the submitted indicators, deleted duplications and collated all indicators into a survey document. This was circulated to the expert advisory group in October 2019 for final comment.

All expert group members were asked to identify colleagues from across South Africa who met the inclusion criteria as panellists for the second phase of the process, Delphi 2. These were (1) South African HCP, including specialist doctors, general practitioners, registered nurses, allied healthcare professionals and social workers who have been trained in PC or who regularly refer patients to PC services, (2) having more than five years’ experience in their relevant speciality or discipline and (3) considered by the expert advisory group to be expert in their field. This tool aims to develop an indicators tool for adults and thus paediatricians were excluded.

The final survey document was sent out via the online anonymous survey tool called REDCAP, a secure web platform for building and managing online databases and surveys, to 116 professionals across South Africa in November 2019. The professions of the panellists are listed in Table 1.

**TABLE 1:** Panellists for Delphi 2.

Profession	Number
Family physicians	11
Geriatricians	13
Emergency medicine physicians	1
Internal medicine physicians	28
Surgeons	16
Gynaecologists	3
Haematologists	7
Registered nurses	12
Social workers	2
PC trained physicians	11

**Total**	**116**

PC, palliative care.

The response rate to the second phase of the process (Delphi 2) was 37% and came from 43 participants across South Africa. Two reminders were sent via email although these had a limited effect on the response rate.

Some participants contributed statements out of keeping with common practice in PC, but these were retained to ensure complete capture of all ideas concerning PC from the surveyed sample. This raised a concern that even experienced HCP may not grasp the role of PC. This further supported the investigators’ intention of providing a tool to identify patients who would benefit from PC, in South Africa.

The responses of the 43 participants were collated and reviewed at a second meeting of the expert advisory group, convened in January 2020. A domain was included if the majority (median ≥ 80%) of the panel had scored it at 8 or more out of a maximum of 10 (1/10 being not for inclusion and 10/10 being for definite inclusion). The domains agreed for inclusion were respiratory, renal, hepatic, neurological, cardiovascular, haematological and infectious diseases and cancer and dementia. The expert advisory group recommended that frailty be added, as in the original SPICT^TM^, as a distinct element alongside dementia.

Domains where agreement was not reached were trauma, mental health and gastrointestinal disease. Most participants scored these at 6–8/10. Trauma, infectious disease and haematological disease were included in the possible list for inclusion based on South African mortality and morbidity data.^13^ Consensus by the expert advisory group was reached that despite the score of 6–8/10, haematological disease should be included because of the global trend to draw attention to the PC needs of such patients who frequently die without PC.^14^

**FIGURE 2:**
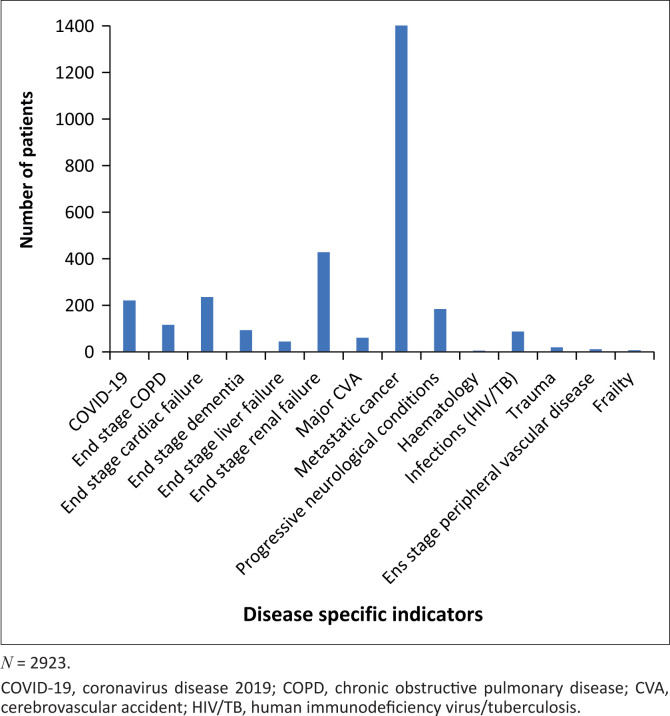
Number of patients with disease-specific indicators referred to a palliative care service.

Even though the general indicators of increasing dependence as formulated by the expert advisory group showed consensus, it was felt that these were too loosely described to be useful. The expert advisory group felt that the tool should however aim to include four to five general indicators. There was consensus by the expert advisory group after another online survey that the developers of the original SPICT^TM^ had already performed a very comprehensive review of these general indicators (Figure 2) and that the original SPICT^TM^ general indicators could be used in the SPICT^TM^-SA. Cognisant of the disparities in healthcare provision and access in South Africa, the expert advisory group felt that it was essential to state clearly, especially in South Africa that ‘this tool is for deteriorating patients with an advanced life-limiting illness where all available and appropriate management for underlying illnesses and reversible complications has been offered’. The expert advisory group felt that disease-specific indicators should be described before the general indicators in the South African indicators tool, so all users of the tool orientate themselves to the disease categories first.

A new draft indicators tool was created taking into account consensus statements and discussions with the expert advisory group. This draft was sent to the 116 panellists via an email link to the REDCap survey in February 2020. There were only 14 responses in this round and no changes were made. This low response rate does limit the findings of this study and further clinical validation will be required to strengthen the validity of this tool. This will be achieved by applying the tool on a patient population and alongside evaluation to determine whether the patients have unmet PC needs.

The new draft indicators tool was externally reviewed by the SPICT programme lead in Edinburgh in Scotland. The Edinburgh group guided the research team, drawing from previous experience in developing and using indicators tools and best practice in adaptation of clinical tools for different settings. This included use of widely accepted language and concepts and the process of consensus building. The final recommended changes were circulated to the expert advisory group for comments after the Edinburgh review.

The original SPICT^TM^ research team has used participatory research to evaluate their tool in clinical practice and concluded that the tool can support clinical judgement by multidisciplinary teams when identifying patients at risk of deteriorating and dying.^3^ The clinical use of the SPICT^TM^-SA can be demonstrated reviewing 2923 PC patient referrals from a database (HREC 012/2016) to a PC service in a tertiary academic hospital. It is evident that people living with cancer remain the most commonly identified patients referred to PC services as indicated in Figure 2 (*n* = 1402). However, clinicians are identifying the need for PC in trauma and infectious diseases as demonstrated by the patients referred to palliative services. The need to include PC in all infectious diseases came to the forefront during the coronavirus disease (COVID) pandemic.^15^

**FIGURE 3:**
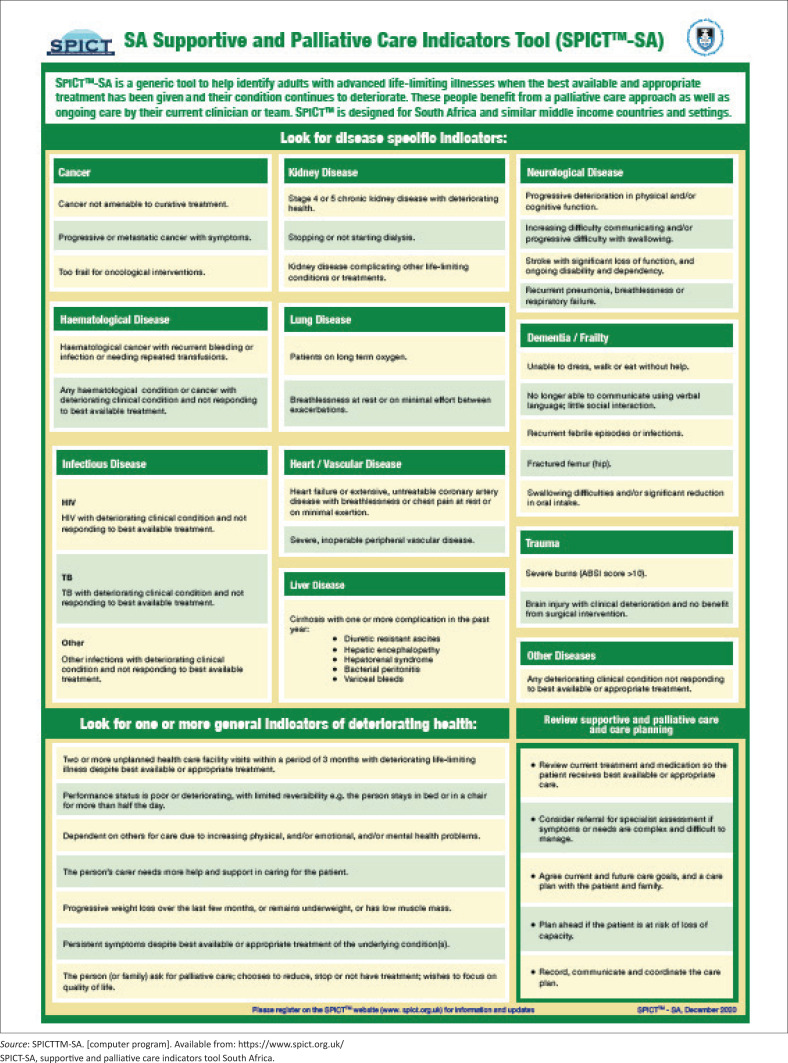
The supportive and palliative care indicators tool South Africa tool.

The final SPICTTM-SA (Figure 3)^16^ developed through the Delphi process will be made available via the SPICT programme website as an open access, downloadable resource.

## Discussion

Implementing the World Health Assembly (WHA) resolution recommendation that all HCPs and health workers should be trained in PC means that all healthcare professionals should integrate PC into the care of patients with life-threatening diagnoses.^1^ This Delphi study aimed to develop consensus on indicators for PC to assist in the early integration of holistic care. A simple tool was created that was named the SPICT^TM^-SA tool. The SPICT^TM^-SA assists HCP by prompting them to recognise which patients need a PC approach integrated with usual care, even when the treatment intent is curative or disease-modifying. The diseases described in the SPICT^TM^-SA include serious illness when patients require optimal management of distressing symptoms, emotional support and discussions relating to advance care planning.

A PC Indicators Tool is a helpful tool to guide healthcare workers to recognise disease specific and general indicators for PC. In developing this SPICT^TM^-SA, there was consensus that general indicators of deteriorating health remain universal across healthcare settings. This tool differs from the original SPICT tool by placing disease-specific indicators before general indicators. Supportive and PC indicators tool South Africa also includes more disease-specific indicators, namely trauma, haematological diseases and infectious diseases.

Contextualisation of disease-specific indicators is required to address the specific disease profile of South Africa and to adapt to the availability of different resources in South Africa. We recognise the limitations of this study in particular the fall off of feedback and input in the later stages of the Delphi process. The low response rate (12%) in Delphi 4 may call into question the validity of the tool. This will be tested during the planned validation study.

Palliative care must never be a substitute for appropriate disease-directed care but rather as supportive therapy that should be offered alongside the best available treatment.^17^ Trauma and infectious diseases are important disease-specific inclusions in LMICs, recognising the morbidity and mortality associated with these conditions.^11^ Haematological conditions need to be included in indicator tools to ensure this population received timely PC. The curative goal in haematological conditions is frequently assessed as being within reach. However, when the condition is no longer responsive to curative treatment, deterioration and death occur rapidly for patients who lack access to PC.^18^

The SPICT^TM^-SA requires validation in the South African and comparable African settings to ensure that it is robust in practical applications and that it accurately identifies patients who would benefit from early PC to reduce health-related suffering because of serious illness. The SPICT^TM^-SA already demonstrates the specific diseases referred to PC services as indicated in the referral pattern to a PC service in a tertiary hospital in South Africa (Figure 2). The development of SPICT^TM^-SA is an iterative process, and it is intended to develop the tool further as clinicians use it and provide feedback. The comments and contributions of users of the SA-SPICT to future improvement would be welcomed (Please provide feedback emailing the main author) - Dr Rene Krause; rene.krause@uct.ac.za.

## Conclusion

The SPICT^TM^-SA is a simple screening tool for South Africa that may assist HCP to recognise patients who may have unmet PC needs. In addition to previously described diseases, this tool includes infectious diseases, trauma and haematologic diseases, which reflects the current South African disease burden.
