# Corrigendum: A Delphi study to guide the development of a clinical indicator tool for palliative care in South Africa

**DOI:** 10.4102/phcfm.v18i1.5566

**Published:** 2026-06-01

**Authors:** Rene Krause, Alan Barnard, Henriette Burger, Andre de Vos, Katya Evans, Lindsay Farrant, Nicki Fouche, Sebastiana Kalula, Jennie Morgan, Zainab Mohamed, Eugenio Panieri, Tasleem Ras, Peter Raubenheimer, Estelle Verburgh, Kirsty Boyd, Liz Gwyther

**Affiliations:** 1 Department of Family Medicine and Public Health, Faculty of Health Sciences, University of Cape Town, Cape Town, South Africa; 2 Department of Oncology, Faculty of Health Sciences, Stellenbosch University, Cape Town, South Africa; 3 Department of Social Work, Groote Schuur Hospital, Cape Town, South Africa; 4 Department of Emergency Medicine, Faculty of Health Sciences, University of Cape Town, South Africa; 5 Department of Nursing, Faculty of Health Sciences, University of Cape Town, Cape Town, South Africa; 6 Department of Medicine, Faculty of Health Sciences, University of Cape Town, Cape Town, South Africa; 7 Department of Oncology, Faculty of Health Sciences, University of Cape Town, Cape Town, South Africa; 8 Department of General Surgery, Faculty of Health Sciences, University of Cape Town, Cape Town, South Africa; 9 Usher Institute, Primary Palliative Care, University of Edinburgh, Edinburgh, United Kingdom; 10 Department of Family Medicine, School of Public Health and Family Medicine, University of Cape Town, Cape Town, South Africa

In the original article published, Krause R, Barnard A, Burger H, et al. A Delphi study to guide the development of a clinical indicator tool for palliative care in South Africa. Afr J Prm Health Care Fam Med. 2022;14(1), a3351. https://doi.org/10.4102/phcfm.v14i1.3351, an author name was incorrectly spelt as Estelle Verburg. The correct spelling is Estelle Verburgh.

The authors apologise for this error. The correction does not change the study’s findings of significance or overall interpretation of the study’s results or the scientific conclusions of the article in any way.

